# Area and Volumetric Density Estimation in Processed Full-Field Digital Mammograms for Risk Assessment of Breast Cancer

**DOI:** 10.1371/journal.pone.0110690

**Published:** 2014-10-20

**Authors:** Abbas Cheddad, Kamila Czene, Mikael Eriksson, Jingmei Li, Douglas Easton, Per Hall, Keith Humphreys

**Affiliations:** 1 Department of Medical Epidemiology and Biostatistics, Karolinska Institutet, Stockholm, Sweden; 2 Human Genetics, Genome Institute of Singapore, Singapore, Singapore; 3 Centre for Cancer Genetic Epidemiology, Department of Public Health and Primary Care, University of Cambridge, Cambridge, United Kingdom; University of Texas School of Public Health, United States of America

## Abstract

**Introduction:**

Mammographic density, the white radiolucent part of a mammogram, is a marker of breast cancer risk and mammographic sensitivity. There are several means of measuring mammographic density, among which are area-based and volumetric-based approaches. Current volumetric methods use only unprocessed, raw mammograms, which is a problematic restriction since such raw mammograms are normally not stored. We describe fully automated methods for measuring both area and volumetric mammographic density from processed images.

**Methods:**

The data set used in this study comprises raw and processed images of the same view from 1462 women. We developed two algorithms for processed images, an automated area-based approach (CASAM-Area) and a volumetric-based approach (CASAM-Vol). The latter method was based on training a random forest prediction model with image statistical features as predictors, against a volumetric measure, Volpara, for corresponding raw images. We contrast the three methods, CASAM-Area, CASAM-Vol and Volpara directly and in terms of association with breast cancer risk and a known genetic variant for mammographic density and breast cancer, *rs10995190* in the gene *ZNF365*. Associations with breast cancer risk were evaluated using images from 47 breast cancer cases and 1011 control subjects. The genetic association analysis was based on 1011 control subjects.

**Results:**

All three measures of mammographic density were associated with breast cancer risk and *rs10995190* (p<0.025 for breast cancer risk and p<1×10^−6^ for *rs10995190*). After adjusting for one of the measures there remained little or no evidence of residual association with the remaining density measures (p>0.10 for risk, p>0.03 for *rs10995190*).

**Conclusions:**

Our results show that it is possible to obtain reliable automated measures of volumetric and area mammographic density from processed digital images. Area and volumetric measures of density on processed digital images performed similar in terms of risk and genetic association.

## Introduction

A mammogram is normally used for detection of breast cancers, either in screening or as part of a clinical work up procedure. In recent years there has been intensive research into the information contained in a mammogram in terms of its value in assisting the prediction of breast cancer risk. Breast tissue density is reflected in the amount of fibroglandular tissue that exists in the breast which appears in mammograms as bright areas. The proportion of the total breast area classified as dense tissue is known as percent density (PD) and has been shown to be a strong determinant of breast cancer risk [1], [2], independently of other established risk factors.

The classical analog mammogram is essentially a film-based projection of the breast which is mechanically scanned in and digitized (i.e., conversion from analog to digital). For digitized analog images there exists a gold-standard for measuring area density, Cumulus [3]. Although it has been widely used in a research context, its use is not feasible on very large studies or in the clinical setting due to it being semi-automated, i.e., user assisted. We have previously developed an automated procedure which mimics this measure [2] and has been validated and used in research [4], [5]. These area-based measures of mammographic density do not take into account the thickness of the dense tissue and rely on a flat two-dimensional projection. Volumetric density can be calculated from screen-film images if a physical phantom, for machine calibration, is placed on the machine’s plate at time of mammography, and if acquisition parameters (APs) are recorded. For analog images, a few studies comparing area- and volumetric-density, in terms of breast cancer risk association, have been reported. Their conclusions, however, differ. Boyd et al. [1] found that measurement of the volume of breast tissue did not improve prediction of breast cancer risk as compared to that using an area-based measure. Aitken et al. [6] concluded that area percent density is a stronger predictor of breast cancer risk than the volumetric method SMF (version 2.2ß), whilst Shepherd et al. [7] concluded that volumetric measures of breast density are better predictors of breast cancer risk than percent dense area.

Recent years have seen a shift from analog to full-field digital mammography (FFDM), in which images are acquired directly with high quality. FFDM images are produced in a raw format which gets altered digitally to yield better visualisation. Both *for processing* (*raw*) and *for presentation* (*processed*) images occupy a large memory size. Typically only processed images are stored in the PACS (picture archiving and communication system) [8]. Although there are now FDA cleared algorithms for determining volumetric density on raw digital mammograms, there are few algorithms that measure mammographic density on processed images; that is, the images normally used in clinical settings. Software that needs the raw data (such as computer-aided detection and volumetric breast density algorithms) processes and then deletes the data [9]. Although the semi-automated Cumulus (area-based) approach has been shown to yield correlated area density measurements from raw and processed images [10]–[13], automating the unification of measurements of processed digital images is a more complicated task than it is on their raw counterparts, owing to the fact that the procedure of processing raw images is not standardised and varies widely by mammography machine vendors who keep the details of their algorithms undisclosed. To use retrospective data sets for mammographic image studies there is a need for algorithms which work well on processed data.

In this article we assess the feasibility of measuring PD from processed FFDM images. We do this using a study in which both raw and processed FFDM images have been collected prospectively. Our approach to volumetric density measurement is based on mimicking Volpara, an FDA cleared algorithm for measuring volumetric density on raw digital mammograms, by combining information on spatial features and acquisition related tags. Additionally, we developed a fully automated approach to measuring area-based density in processed digital images. We compare measurements derived from processed images using our approaches to those obtained from Volpara on corresponding FFDM raw images, directly and in terms of association with breast cancer risk and with the genetic variant, *rs10995190*. All of these comparisons are important and it can even be argued that calibrating density measures against a genetic variant is preferable to using disease status [14]. We also study the importance of individually extracted image textural features on breast cancer risk and the genetic variant. To the best of our knowledge this is the first article to compare volumetric and area based density on FFDM images in terms of their association with breast cancer risk or a genetic variant for breast cancer/mammographic density. Moreover, we believe that this work forms the first attempt to generate volumetric mammographic density from processed FFDM images without the need for a calibration reference phantom.

## Materials and Methods

### Main study population

All women included in the current study participate in the ***Kar*** *olinska* ***Ma*** *mmography* cohort (KARMA) study (http://karmastudy.org/), which is a prospective cohort study that was initiated in January 2011 and comprises women attending mammography screening or clinical mammography at four hospitals in Sweden. Upon study entry, participants donated blood and filled out a detailed web-based questionnaire. In addition, permission was asked for storage of both raw and processed FFDM and linkage to Swedish national registers on inpatient care and cancer. The main analysis presented in this article is based on 47 women diagnosed with incident breast cancer (in KARMA) and 1011 healthy women (in KARMA) with FFDM images (raw and processed, medio-lateral oblique (MLO) images) for the GE Medical Systems, *model:* Senographe Essential version ADS 53.40 and *station name:* HBGMG03. For the 1011 healthy women we have genotype data from iCOGs, a custom illumina iSelect genotyping array designed for replication and fine mapping of common and rare variants with relevance to breast, ovary and prostate cancer [15]. We also had access to the same types of images for an additional 403 healthy women in KARMA (genotype data is not available for these women). Their images were used for developing/training our automated volumetric density measure, CASAM-Vol, to predict Volpara, in a step carried out prior to performing the analysis which used images from the 47 and 1011 women. For the 47 women with breast cancer, all images included in analyses presented here are from the contralateral breast and were taken prior to diagnosis (but less than 3 years prior to diagnosis). For the 1414 healthy women we selected the LMLO view of the image.

### Questionnaire data

Information on age, BMI, hormone replacement therapy (HRT) status, reproductive history and other breast cancer risk factors were collected via a web-based questionnaire at study entry. Menopausal status was defined according to information on last year menstruation status, previous oophorectomy and age at study entry.

### Ethics Statement

The Karma study has an ethical committee approval by the Ethical Committee at Karolinska Institutet (Dnr 2010/958-31/1) and all participants provided written informed consent.

### Measures of PD compared in this study

#### Volpara (for raw images, version: 1.4.3|3433|)

Volpara is an FDA cleared algorithm for measuring volumetric density on both 2D medio-lateral oblique (MLO) and 2D cranio-caudal (CC) raw digital mammograms. The software makes use of the physics information stored with digital mammograms (the acquisition parameters) to work backwards from the pixel intensity values in the raw image to the X-ray attenuation between the pixel and the X-ray source, and uses the X-ray attenuation of an entirely fatty region as an internal reference; see [16], [17]. Volumetric percent density is obtained as the ratio of dense volume and breast volume, where dense volume is obtained by summing the dense thickness across all pixels in the breast and breast volume is obtained by multiplying the breast area by the recorded compressed breast thickness, with a correction for the breast edge. By design, Volpara is not extendable to work on processed FFDM or on digitised film mammogram images. That is, its algorithm assumes that pixel values are proportional to exposure, which is not the case for processed images since the pixel values are non-linearly transformed to enhance the contrast [8]. It is important to know that manufacturers keep the raw-to-processed conversion algorithm secret. A blind reverse engineering of this conversion is not feasible due to non-linearity of the transformation which may be also assisted by some acquisition parameters. Volpara’s generated PD is measured on a volumetric scale which is lower than that of area-based PD measures. The approach has recently been demonstrated to correlate well with density measured from magnetic resonance imaging (MRI) images [9], [16], [17] and a visual assessment method, Breast Imaging-Reporting And Data System (BIRADS) [17]. We use a natural log transformation of Volpara in all analyses presented here which results in values being roughly symmetrically distributed. To date, there have only been a few other attempts to derive volumetric density measures from FFDM images. Heine et al. [18], for example, quantified PD from raw CC images after performing calibration to adjust for inter-image acquisition technique differences.

#### CASAM-Area (for processed images)

CASAM is an acronym for Computer Aided Statistical Assessment of Mammograms. The second density measure assessed here is our own measure of area-based PD on processed mammograms obtained from directly segmenting the breast and fibro-glandular dense tissue areas in the two dimensional space. We have carefully designed our pre-processing steps (of the processed images) in order to arrive at a reliable PD measurement and thereafter to extract meaningful statistical features. In the pre-processing phase we utilised contrast limited adaptive histogram equalization (CLAHE). CASAM-Area takes the square-root of an area-based PD measure. It is calculated as
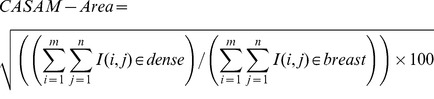
(1)where *m* and *n* are the dimensions of the image *I* and *dense* and *breast* refer to segmented regions in the image; see the supplementary material for detailed information about the method.

*CASAM-Vol (for processed images)*: CASAM-Vol is obtained as a weighted combination of statistical and morphological features (measured in processed images) and acquisition related tags, with weights obtained by training a random forest, an ensemble learning nonparametric statistical method for classification and regression developed by Breiman [19], to predict *log* Volpara measurements (from raw images). Images for the subset of 403 women were used for training. The acquisition parameters and statistical/morphological features used for training against Volpara are described in more detail below. We used the pre-compiled MATLAB mex-files [20] to find the optimal value of the shrinkage tuning parameter in which the number of trees was set to 500. Weights obtained from the training data set were subsequently used to produce CASAM-Vol measures, i.e. predicted values of log Volpara, for the processed images from the independent subsets of 47 and 1011 women (the test data). The acquisition parameters, extracted from the image *header*, which we used as inputs to the random forest were: *KVP, XTC (ExposureIn µAs/ExposureTime), ExposureTime, X-rayTubeCurrent, Exposure, ExposureIn µAs, BodyPartThickness, Log(ExposureIn µAs), (1/BodyPartThickness), log(BodyPartThickness), (1/ExposureIn µAs), CompressionForce, AnodeTargetMaterial, RelativeXrayExposure and OrganDose*. We chose to use the acquisition parameters (in addition to features measured in processed images) since they are incorporated into Volpara and are used in the volumetric percent density measure described by [1].

The computer-aided procedure for extracting the features which were used in training CASAM-Vol is described in the supplementary material (see, Algorithm S1). The algorithm for pre-processing the processed images was developed to reveal structures within the breast and to lessen the mal-effect of contrast intensity fluctuation. After pre-processing each processed image we segmented the breast region and detected and removed the pectoral muscle. For intra-breast segmentation, uniform thresholding clearly entails knowledge of the intensity values’ range, otherwise regions of interests may be missed in the selection process [21] Ch. 3, p. 94. We used a multi-thresholding approach as has been used in [2]. To help circumvent the dense-fatty low contrast issue we apply background subtraction by subtracting the morphologically open image (with a disk-shaped structuring element with a radius of 50 pixels) from its original image. We then applied 7 thresholding methods to obtain cut-offs from which 12 regions within the breast were determined; see Table S2 in the supplementary material. Finally, a variety of low level and high level features were evaluated comprising 55 measurements for each mammogram (in most cases recalculated for each of the 12 regions, resulting in a feature vector of length 489). The 55 measurements are listed in Table S1.

### Genetic data

For the subset of women with iCOGs data we used genotypes for the SNP *rs10995190*, in the gene *ZNF365*. This SNP has been confirmed to be associated with both mammographic density (p = 9×10^−10^) [22] and breast cancer risk (p = 1×10^−36^) [15].

### Statistical analysis

To evaluate association between each of the automated PD measures and genotypes of the SNP *rs10995190* (coded 0/1/2, treated as continuous variable), we fitted linear regression models using PD measures one at a time as outcome variables and carried out Wald tests. For CASAM-Area we used the square-root transformation, as in [2], [22], to obtain a variable which follows an approximate normal distribution. For Volpara measurements we used a logarithm transform since the distribution of the untransformed measurements are more heavily skewed than the area based measures. In addition to genotype, each model included the variables age (in years) and BMI, menopausal status, HRT use, parity and age at first birth as covariates. We also carried out similar tests of association for each PD measure additionally adjusting for the other PD measures (one at a time).

To explore whether the textural features in Table S1, and the acquisition parameters listed earlier, could be independently associated with *rs10995190* we carried out further association tests (again by fitting regression models). We first carried out tests adjusting for age, BMI, menopausal status, HRT use, parity and age at first birth, and subsequently additionally adjusting for one other area- or volumetric-PD measure. Each feature was transformed using a Box-Cox transformation (using the *R* package MASS; [23]). **–**Log_10_ p-value QQ plots were constructed to summarise the results of these tests using the *R* package Haplin [24]. We also performed a global test of association testing the null hypothesis that none of the features are associated with *rs10995190* after adjusting for PD (Volpara), age and BMI. We first calculated the residuals from fitting regression models with each feature as an outcome (one at a time) and Volpara, age and BMI as covariates. To account for the correlation structure of the features we carried out a permutation (global) test, using as a test statistic the number of p-values<0.05 from testing association (using a Wald test from a linear regression model) between the residuals and genotypes. We first did this for the observed data and for 10,000 data sets with the genotypes permuted. To obtain our global p-value we compared the value of our global test statistic from the observed data to the distribution of the test statistic from the permuted data sets.

In addition to the genetic association analyses described above we studied the association between the density measures and breast cancer. After combining the data from the subsets of 47 and 1011 women, we evaluated the association between case-control status and the different PD measures using unconditional logistic regression (case/control status as dependent variable and each of the features as the independent variable). As well as adjusting for all potential confounders (used in the genetic analysis), we also carried out analyses with partial adjustment (for age and BMI). We did this because of the small number of cases and to avoid over-adjustment. Finally, we carried out tests of association for each PD measure adjusting for each of the other measures (one at a time) and tested for association with the textural features in Table S1 (using unconditional logistic regression).

R (version 2.13.0) was used for data management, statistical analyses and graphics [25]. All reported tests are two-sided. All of the models were adjusted for age, BMI, menopausal status, HRT use, parity and age at first birth.

## Results

Characteristics of the women and their mammographic images, included in each of the data subsets are described in Table 1. No significant differences between the genetic association (control) data set and the cases were observed for the APs, Volpara, CASAM-Area and CASAM-Vol. Differences in age, menopausal status and HRT use were observed but these factors were adjusted for in the case-control analysis (below).

**Table 1:** Key characteristics of individuals included in this study (mean (s.d) or n (%)).

	Data used for developing CASAM-Vol (n = 403)	Data used for genetic association study (*) (n = 1011)	Cases used for case-control study (n = 47)	P-value (ψ), (**)(comparing columns 3&4 data sets)
**Age**	55.93 (9.12)	53.52 (9.45)	58.59 (8.43)	<0.001
**BMI**	25.12(4.17)	25.92 (4.46)	25.67 (4.27)	0.710
**Postmenopausal**				0.003
No	148 (37)	508 (50)	33 (69)	-
Yes	240 (60)	477 (47)	14 (29)	-
**HRT use**				0.041
Never	241 (60)	707 (70)	35 (73)	-
Past	102 (25)	204 (20)	6 (13)	-
Current	30 (7)	42 (4)	6 (13)	-
**Parity and age at first birth**				0.286
Nulliparous	49 (12)	101(10)	9 (19)	-
Parity ≤2 and age at first birth ≤25	95 (24)	250 (25)	13 (27)	-
Parity ≤2 and age at first birth >25	131(33)	380 (38)	16 (33)	-
Parity >2 and age at first birth ≤25	78 (19)	154 (15)	7 (15)	-
Parity >2 and age at first birth >25	35 (9)	99 (10)	2 (4)	-
**Acquisition Parameters (APs)**				
KVP	29.11 (1.17)	29.44 (1.07)	29.34 (1.13)	0.332
XTC	68.73 (14.75)	66.28 (12.40)	68.57 (14.76)	0.184
ExposureTime	721 (252.86)	819 (316)	767 (293)	0.230
XRayTubeCurrent	68.12 (14.66)	65.68 (12.39)	68.14 (14.82)	0.175
Exposure	47.42 (13.40)	52.59(18.03)	50.37 (15.89)	0.390
ExposureInMicroAs	47403 (13388)	52574 (18023)	50353(15844)	0.377
BodyPartThickness	58.53 (14.53)	61.43 (14.03)	59.02 (14.04)	0.232
AnodeTargetMaterial	0.823 (0.38)	0.884 (0.32)	0.823 (0.38)	0.195
RelativeXrayExposure	4748 (1746)	5426 (2280)	5116 (2025)	0.329
OrganDose	0.010 (0.0022)	0.011 (0.0031)	0.011 (0.0027)	0.277
CompressionForce	102 (29.69)	101 (30)	106 (27.99)	0.209
**Volpara (raw)**	2.01 (0.56)	2.01 (0.58)	2.12 (0.60)	0.159
**CASAM-Area (Processed)**	4.58 (1.02)	4.57 (1.03)	4.78 (1.01)	0.160
**CASAM-Vol (Processed)**	2.01 (0.49)	2.01 (0.50)	2.08 (0.51)	0.393

(*) Also used as controls in the case-control study. (**ψ**) Wald test p-values (logistic regression, unadjusted). (**) LR tests for menopausal status, HRT use and parity.

**Figure 1:**
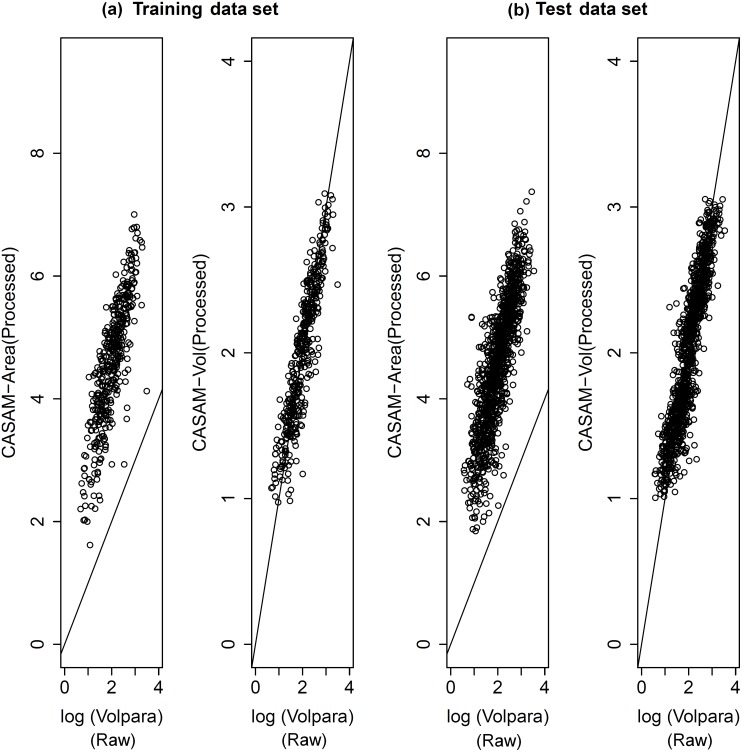
Correlation between Volpara and our PD measurements from processed mammograms: (a) Scatter-plots of our PD measurements (processed mammograms) and Volpara (raw mammograms) measurements for the training sample of GE mammograms from 403 women (b) scatter-plots of our PD measurements and Volpara measurements for the test sample of GE mammograms from 1011 women with genotype information.

We developed/trained our measure of volumetric density (for processed digital images) using the subset of (raw and processed) images for the 403 women. We also measured area PD in their processed images. Scatter plots of these two sets of measurements against Volpara measurements from corresponding raw images for this training data set are shown in Figure 1 (a). The Pearson correlation coefficients between Volpara and CASAM-Area, and between Volpara and CASAM-Vol, were 0.77 and 0.91, respectively.

We then examined the correlation between our measures of PD from processed digital images with Volpara PD measurements (taken from corresponding raw FFDM images), in the subset of 1011 healthy women; see Figure 1 (b). The Pearson correlation coefficients between Volpara and CASAM-Area, and between Volpara and CASAM-Vol, were 0.84 and 0.91, respectively. From both the training (n = 403) and test (n = 1011) data sets plots, we can assert that it is possible to obtain a reliable volumetric mammographic density from processed images based on predicting Volpara values. Although CASAM-Area and Volpara measures differ conceptually, the correlation between them was observed to be fairly strong (r = 0.84).

We next assessed the association between the SNP *rs10995190* and each of the automated PD measures, using our test data sets. All three automated PD measures were associated with *rs10995190*, after adjusting for age, BMI, menopausal status, HRT use, parity and age at first birth (p<1×10^−6^); see Table 2. As soon as one measure of density was adjusted for, the other measures were, at best, weakly associated with *rs10995190*. There was some evidence that the area and volumetric based approaches complement each other, but only to a very small extent; p = 0.036 for the Volpara – *rs10995190* association, after adjusting for CASAM-Area, and p = 0.047 for the CASAM-Area – *rs10995190* association after adjusting for Volpara. CASAM-Vol (from processed images) appeared to mimic well Volpara (from raw images) (p = 0.079 for the test of “residual” association in Table 3).

**Table 2:** Effect estimates for *rs10995190* on automated measures of mammographic density.

Outcome	Estimate (95%CI)	p-value
Volpara (raw)	–0.138(–0.191, –0.085)	4×10^−7^
CASAM-Area (Processed)	–0.254(–0.353, –0.155)	6×10^−7^
CASAM-Vol (Processed)	–0.113(–0.158, –0.068)	9×10^−7^

Point estimates, interval estimates and p-values (Wald tests) are based on estimated coefficients for the SNP in linear regression models with PD measures as outcomes, adjusting for potential confounding variables (n = 1011).

**Table 3:** p-values assessing the association of the automated measures of mammographic density with *rs10995190* (after additional adjustment for one other density measure) (n = 1011).

Outcome variable	Variables adjusted for			
	Standard^(^*^)^	Standard + Volpara	Standard + CASAM-Area (Processed)	Standard + CASAM- Vol
Volpara	4×10^−7^	-	0.036	0.079
CASAM-Area (Processed)	6×10^−7^	0.048	-	0.147
CASAM-Vol (Processed)	9×10^−7^	0.198	0.282	**-**

(*) adjusting for age, BMI, menopausal status, HRT use, parity and age at first birth.

**Figure 2:**
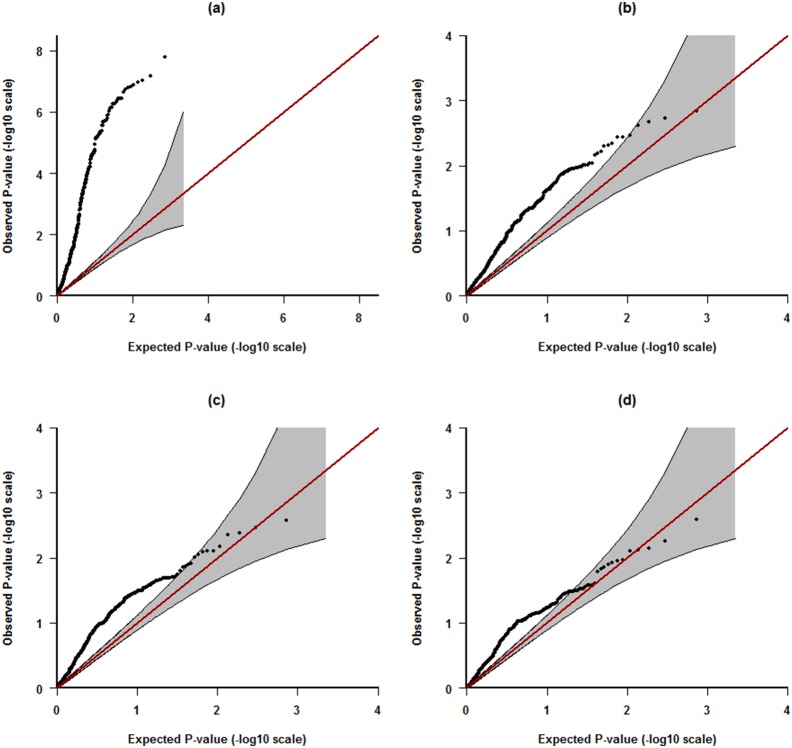
–log_10_ QQ plots for p-values assessing the association between the investigated features (Table S1 and APs) and *rs10995190*. (a) with adjustment for age, BMI, menopausal status, HRT use, parity and age at first birth. (b) with adjustment as in *(a)*, plus Volpara. (c) with adjustment as in *(a)*, plus CASAM-Vol. (d) with adjustment as in *(a)*, plus CASAM-Area.

We next evaluated association between each of the statistical/textural features and each of the percent density measures. The QQ-plot in Figure 2 (a) shows that the features, as a whole, are strongly associated with *rs10995190* without adjusting for a measure of PD (i.e. with *standard* adjustment for age, BMI, menopausal status, HRT use, parity and age at first birth). After adjusting additionally for Volpara, there remained some evidence of association (Figure 2 (b)). The QQ plots summarising association tests based on adjusting instead for CASAM-Area (Figure 2 (c)) and CASAM-Vol (Figure 2(d)), were similar to those based on adjusting for Volpara. Since the association tests summarised by the QQ-plots are correlated (many of the features and APs are correlated), for the tests based on adjusting for Volpara, we carried out a permutation test of a global null hypothesis (none of the features are associated). We obtained a p-value of 0.047, suggesting that there is some useful information in the images which is not captured by Volpara.

Subsequently, we evaluated the association of PD measurements with cancer risk (case/control status). Table 4 summarises results from fitting logistic regression models with breast cancer status as outcome and PD measurements (one at a time) as a covariate, along with other potential confounders.

**Table 4:** Effect estimates for automated measures of mammographic density on case-control status, n = 1058 (Cases 47, Controls 1011).

Covariate	Estimate (95%CI)	p-value
**(a)**		
Volpara(raw)	0.978 (0.300, 1.660)	0.005
CASAM-Area (Processed)	0.483 (0.112, 0.862)	0.012
CASAM-Vol (Processed)	0.926 (0.124, 1.730)	0.023
**(b)**		
Volpara(raw)	0.961 (0.239, 1.706)	0.010
CASAM-Area (Processed)	0.467 (0.071, 0.879)	0.023
CASAM-Vol (Processed)	0.813 (–0.041, 1.691)	0.065

Point estimates, interval estimates and p-values (Wald tests) are based on estimated coefficients for PD in logistic regression models with case-control status as outcome. (a) with partial adjustment (age and BMI), (b) with full adjustment (age, BMI, menopausal status, HRT use, parity and age at first birth).

After adjusting for one measure of PD, none of the other measures were significantly associated with case-control status (p>0.10; Table 5). We tested for association with each of the features listed in Table S1, and each of the APs. The QQ plot summarising the tests which adjusted for Volpara, showed no evidence of association (i.e., no significant deviation from the 45^o^ line) between the features/APs and case-control status (data not shown).

**Table 5:** p-values assessing the (residual) association between automated measures of mammographic density and case-control status (after adjustment for one other density measure).

Covariates	Variables adjusted for			
	Standard	Standard + Volpara	Standard + CASAM-Area (Processed)	Standard + CASAM- Vol
**(a)**				
Volpara	0.005	-	0.479	**0.823**
CASAM-Area (Processed)	0.012	0.162	-	**0.960**
CASAM-Vol (Processed)	0.023	**0.088**	**0.235**	**-**
**(b)**				
Volpara	0.010	-	0.529	**0.561**
CASAM-Area (Processed)	0.023	0.172	-	**0.769**
CASAM-Vol (Processed)	0.065	**0.054**	**0.158**	**-**

(a) with partial adjustment (age and BMI), (b) with full adjustment (age, BMI, menopausal status, HRT use, parity and age at first birth) (n = 1058).

## Discussion

We found the CASAM-based mammographic density measurements to be associated with breast cancer status and *rs10995190* (*ZNF365*), with amount of evidence similar to that found for volumetric measure in raw images (Volpara), suggesting that it is possible to measure density in an automated fashion using processed FFDM images. The p-values from tests of genetic association for the volumetric and area measures were observed to be similar. We found some evidence to suggest that area and volumetric measures of density can complement each other. Our case-control analysis, based on 47 cases, was not able to show that area and volumetric measures of density can complement each other in risk prediction probably due to lack of power. If this can be shown, statistics integrating area and volumetric density should be developed (see [14]). For risk prediction, larger studies are needed to address whether volumetric approaches to breast density measurement in two-dimensional digital images can offer gains over standard area-based measures. Even for analog images it is unclear whether volumetric approaches are markedly better than area approaches [26]. Shepherd et al. [7], however, used digitized film mammograms (275 cases and 825 controls) matched for age, ethnicity, and mammography system, assessed three measures of breast density: PD, fibroglandular volume, and percent fibroglandular volume, and did conclude that volumetric measures of breast density provide more accurate predictors of breast cancer risk than area-based PD.

Approaches for measuring volumetric PD are typically based on calibrating a model against a phantom. Heine et al. used a balloon filled up with water (mimicking the fatty region in a breast) and oil (mimicking the dense region) [27]. Boyd et al. created plastic phantoms representing a range of combinations of fat/fibroglandular tissue to calculate the volumetric percentage density [1]. They based their study on digitized analog films (16 machines in 7 different locations in Canada), all images were CCs (364 cases/656 controls). Breast thickness was recorded under different compression forces along with the thickness read by the machine. Their hypothesis was that the breast thickness reported by a mammography machine needs correction since compression is such that the two used plates will not be perfectly parallel. Additional corrections for exposure and processing were made using a step wedge included in each image. The authors concluded, however, that measurement of the volume of breast tissue, based on utilising APs, in two-dimensional images, did not improve prediction of breast cancer risk over area-based measures. Other researchers have also compared volumetric and area-based measures of PD in two-dimensional images. Ding et al. [28] carried out a large case-control study comprising 634 cases with 1,880 age-matched controls. They used the standard mammography form (SMF) technique to verify the association of the volume of breast density with risk of breast cancer and to compare these measurements with Cumulus readings. SMF uses information about the thickness of the compressed breast, tube voltage and exposure time, to estimate the breast tissue volumes. These volumes were associated with breast cancer risk but less strongly so than the measured area PD (note that Volpara represents an improved version of SMF [13]).

It is possible that the density measures studied in the paper are unable to capture every aspect of density completely. This is supported, in our data, by the fact that the association between rs10995190 and statistical/textural features does not completely disappear after adjusting for these PD measures. No single feature sticks out from the others in terms of its association with *rs10995190* and furthermore it is difficult to interpret individual statistical/textural features in mammographic images [29]–[31]. The features could relate directly to some biological change in breast composition but, on the other hand, these features could be capturing some aspect of the X-ray energy that is a proxy for breast composition or dense tissue thickness.

It is clear from Figure 1 that CASAM-Area has a narrower range of values than the conventional area-based methods. This is an inevitable phenomenon since the algorithm encompasses the use of a histogram equaliser called contrast limited adaptive histogram equalization (CLAHE). Applying CLAHE to the mammogram images has significantly increased the accuracy of our algorithm in picking up the dense region within the breast area. This was helpful because CLAHE’s underlying algorithm, which is well adopted in medical imaging field, uses a sophisticated adaptive process to enhance image contrast without any saturation occurrences. The reason behind CASAM-Area’s lower PD range in Figure 1 being truncated is that CLAHE operation increases the signal-to-noise ratio while highlighting dim structures, in our case that refers to blood vessels in a fatty breast which are classified by our algorithm as dense tissue as one can easily identify by examining Figure S1 (b). On the other hand, the truncation shown in CASAM-Area’s upper PD range in Figure 1 could be due to the fact that in a very dense breast, the dense region can be optically exaggerated; making it difficult to distinguish the real dense area border because of the fuzzy gradient contour that arise from an optical occurrence known as the point spread function. By virtue of the properties of CLAHE, the effect of the point spread function is greatly diminished. We believe that our pre-processing and segmentation steps are important for the success of our algorithm, which is why we describe these key steps in some technical details in the supplementary material to provide clarity and to ease replication.

Inclusion of the analysis of association using the genetic variant is a strength of the analysis because case-control association analysis of mammographic images is theoretically susceptible to bias, if there are differences in mammography machines used between cases and controls [14]. For calibrating density measures, it may be better to use genetic variants of mammographic density and breast cancer risk than breast cancer status.

Volpara is cleverly designed to reproduce the volume of breast composition from a 2D projection with high accuracy. However, it works only on raw images and for processed digital images there is no established fully-automated method for measuring density. Although the medical and scientific community are slowly picking up on the value of storing raw images, there are, to date, huge archives of processed digital images which stand to benefit from retrospective assessment of mammographic density for epidemiologic research. Until prospective studies with data from raw images mature, interim measures such as those described in this article could play a vital role in research.

The results of our association analyses using processed FFDM images were similar to those using raw FFDM images, suggesting that processed images may be viable for large-scale epidemiologic research. In this manuscript we have studied three automated measures of mammographic density. The images included here have not been read by a subjective or semi-automatic method. We note, however, that we find that the association between Volpara and Cumulus (an established percent density semi-automatic method) has previously been reported to be high [13] and that Volpara has also been shown to be strongly associated with MRI density measurements (r  = 0.93) [9]. Quantra (an earlier version of Volpara) has been shown to be associated with the BIRADS classification (89.0% correct classification) [32], and in another recent study Volpara density classification and radiologist’s BIRADS showed a positive strong correlation (r = 0.87; p<0.001) [33].

We expect that the PD measures for processed FFDM images, presented here, will perform consistently for mammograms taken from GE machines. There will, however, inevitably be some variability across different vendor machines due to discrepancies in raw-to-processed conversion algorithms. To address this issue, in cohorts comprising mammograms from different vendor machines, it may be necessary to retrain CASAM-Vol to mimic Volpara on each specific machine. Note that CASAM-Vol is constructed from features which include various acquisition parameters that are also exploited by Volpara and are X-ray system dependent (i.e., are not affected by the raw-to-processed conversion algorithm). We expect that CASAM-Vol can mimic Volpara with similar accuracy across different machines. Brand *et al.* [34] have shown that there are only small differences in distributions of Volpara measurements across different vendor machines. It will therefore probably not be crucial (but it could still be wise) to adjust for machine in case/control or genetic association analyses based on CASAM-Vol. CASAM-Area is more likely, than CASAM-Vol, to be affected by raw-to-processed conversion variability. Like all threshold based methods in the literature, CASAM-Area is image intensity dependent. This intensity is greatly manipulated in an unpredictable way by the raw-to-processed conversion algorithms. For CASAM-Area it will be important to adjust for machines when fitting statistical models in studies incorporating multiple mammography machines.

## Supporting Information

Figure S1 **Pre-processing of mammograms: (a) original mammograms, (b) pseudo-colour generation after applying the horizontal and vertical cropping, (c) the positive signal in the** ***Q (x,y)*** **colour space (** ***Q (x,y)>0*** **), detecting the reddish area (d) convex hull of the negative** ***(c)*** **, (e) the final extracted breast mask, and (f) breast region after applying the contrast limited adaptive histogram equalization (CLAHE).**
(TIF)

Table S1 **The derived statistical and textural features.**
(DOC)

Table S2 **The table depicts the twelve different regions used in our approach from which features in Table S1 are derived.**
(DOC)

Algorithm S1 **A detailed description of the underlying algorithm for mammography image processing and segmentation.**
(DOCX)

Data S1 **Data for Analyses is an Excel CSV file containing the data set we used in this study.**
(CSV)

File S1 **RCode is a text file which accesses the “** ***Data for Analyses*** **” file to regenerate our results.**
(TXT)
