# Supplementary figures and images for: Area and Volumetric Density Estimation in Processed Full-Field Digital Mammograms for Risk Assessment of Breast Cancer
Source: PLoS One. 2014 Oct 20;9(10):e110690. doi: 10.1371/journal.pone.0110690 (PMC4203856; doi:10.1371/journal.pone.0110690)

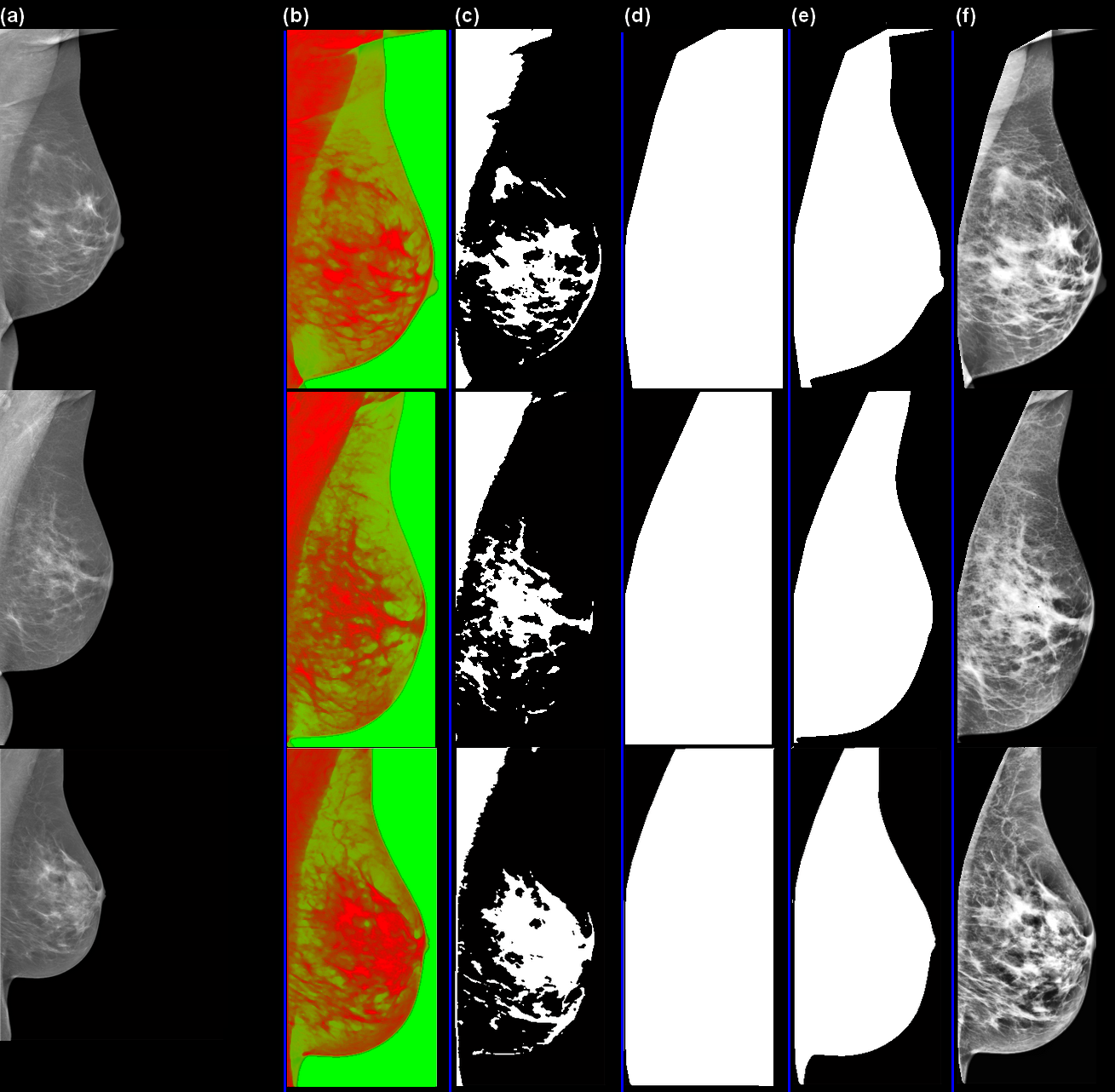

Supplement: Figure S1 — Pre-processing of mammograms: (a) original mammograms, (b) pseudo-colour generation after applying the horizontal and vertical cropping, (c) the positive signal in the Q (x,y) colour space ( Q (x,y)>0 ), detecting the reddish area (d) convex hull of the negative (c) , (e) the final extracted breast mask, and (f) breast region after applying the contrast limited adaptive histogram equalization (CLAHE). (TIF) [file pone.0110690.s001.tif]
