# Supplementary material for: Area and Volumetric Density Estimation in Processed Full-Field Digital Mammograms for Risk Assessment of Breast Cancer
Source: PLoS One. 2014 Oct 20;9(10):e110690. doi: 10.1371/journal.pone.0110690 (PMC4203856; doi:10.1371/journal.pone.0110690)
Supplement: Table S1 — The derived statistical and textural features. (DOC) [file pone.0110690.s002.doc]

Table S1: The derived statistical and textural features

| **Feature** | **Intensity based** | **Morphology/Shape based** |
| --- | --- | --- |
| **PD: Percent density (CASAM-Area)** |  | ● |
| *f1*: The DC value of the 2D discrete cosine transform | ● |  |
| *f2*: The coefficient of the last decomposition of single-level discrete 2D wavelet transform | ● |  |
| *f3*: The max coefficient of the 2D discrete Fourier transform of the local range filter | ● |  |
| *f4*: Kurtosis of the region of interest (ROI)intensity | ● |  |
| *f5*: Skewness of the ROI intensity distribution | ● |  |
| *f6*: Entropy of the ROI intensity | ● |  |
| *f7*: Mean of the entropy filtered ROI | ● |  |
| *f8*: Entropy of the complex imaginary part of the convolved region with log-Gabor filters | ● |  |
| *f9*: Entropy of the complex real part of the convolved region with log-Gabor filters | ● |  |
| *f10*:Entropy of the magnitude part of the convolved region with log-Gabor filters | ● |  |
| *f11*:The max coefficient of the 2D discrete Fourier transform applied to the ROI | ● |  |
| *f12*:Entropy of the normalised co-occurrence matrix of the ROI | ● |  |
| *f13*:The magnitude of the difference mean of the region and the mean of the complement ROI | ● |  |
| *f14*:The max coefficient of the 2D discrete Fourier transform of the Hessian filter applied to the ROI | ● |  |
| *f15*:Entropy of the entire breast area***** | ● |  |
| *f16*:The max coefficient of the log of the magnitude part of the discrete cosine transform applied to the ROI | ● |  |
| *f17*:The max coefficient of the log of the magnitude part of the discrete Fourier transform applied to the ROI | ● |  |
| *f18*:The 4th central moment of the ROI |  | ● |
| *f19*:Number of particles within the ROI |  | ● |
| *f20*:Solidity of the ROI |  | ● |
| *f21*:Eccentricity of the ROI |  | ● |
| *f22*:Euler Number of the ROI |  | ● |
| *f23*:Number of particles within the entire breast***** |  | ● |
| *f24*: (Skewness of the normalised singular value decomposition of the ROI) / (standard deviation of the ROI) | ● |  |
| *f25*:Singular value decomposition of the ROI | ● |  |
| *f26*:Euler number within the breast (binary image) | ● |  |
| *f27*:Interquartile range- the difference between the 75^th^ and the 25^th^ percentiles of the intensity values of the ROI | ● |  |
| *f28*:The 1^st^ Fractal descriptor of the entire breast***** | ● |  |
| *f29*:Mean intensity of the breast***** | ● |  |
| *f30*:Skewness of the eroded ROI intensity | ● |  |
| *f31*:Skewness of the intensity distribution of the breast***** | ● |  |
| *f32*:Kurtosis of the projection along the Y axis of the ROI |  | ● |
| *f33*:Kurtosis of the projection along the X axis of the ROI |  | ● |
| *f34*:Mean perimeter of the ROI |  | ● |
| *f35*:Mean circularity of smallest particles 1-100 pix***** |  | ● |
| *f36*:Mean intensity of the ROI | ● |  |
| *f37*:Homogeneity of the breast area***** | ● |  |
| *f38*:Median intensity of the ROI | ● |  |
| *f39*:Skewness of the gradient of the intensity within the ROI | ● |  |
| *f40*:Energy property of the co-occurrence matrix of the selected area - horizontal shift (2 pixels) | ● |  |
| *f41*:Energy property of the co-occurrence matrix of the selected area - diagonal shift (8 pixels) | ● |  |
| *f42*:Variance of the intensity within the breast area***** | ● |  |
| *f43*:Mean of the local binary pattern (LBP) of the whole breast region***** |  | ● |
| *f44*:Kurtosis of the local LBP histogram of the whole breast region***** |  | ● |
| *f45*:Mean intensity of the pectoral muscle***** | ● |  |
| *f46*:RunLengthCode: Short Run Emphasis (SRE), Long Run Emphasis(LRE), Gray Level Non-Uniformity (GLN), Run Percentage (RP), Run Length Non-Uniformity (RLN), Low Gray Level Run Emphasis (LGRE), High Gray Level Run Emphasis (HGRE)***** | ● |  |
| *f47*:Entropy of the sum of the average values of co-occurrence matrix of the breast along the horizontal and diagonal directions ***** | ● |  |
| *f48*:Standard deviation of FD (fractal descriptor) for the selected area ***** | ● |  |
| *F49:* Lacunarity of FD for the selected area ***** | ● |  |
| *f50:* Laws Texture Energy Measures: Entropy Level, Entropy Edge, Entropy Spot, Entropy Ripple ***** | ● |  |
| *f51:* Microcalcifications ***** |  |  |
| *f52:* Intensity ratio(dense/breast) ***** | ● |  |
| *f53:* Dense area size ***** |  | ● |
| *f54:* Breast area ***** |  | ● |
| *f55:* Percentage Fatty ***** |  | ● |

(*) denotes features derived from a single region, the rest are derived from the 12 regions.
