# Supplementary material for: Area and Volumetric Density Estimation in Processed Full-Field Digital Mammograms for Risk Assessment of Breast Cancer
Source: PLoS One. 2014 Oct 20;9(10):e110690. doi: 10.1371/journal.pone.0110690 (PMC4203856; doi:10.1371/journal.pone.0110690)
Supplement: Table S2 — The table depicts the twelve different regions used in our approach from which features in Table S1 are derived. (DOC) [file pone.0110690.s003.doc]

Table S2. The table depicts the twelve different regions used in our approach from which features in Table S1 are derived.

| **Region Label** | **Description** |
| --- | --- |
| Region 1 | Finds the *dense* region in mammographic images using the *p-tile method* |
| Region 2 | Finds the *dense* region in mammographic images using the *entropy of the histogram*. |
| **Region 3 (*)** | Finds the *dense* region in mammographic images usingthe *moment preserving thresholding method* |
| Region 4 | Finds the *dense* region in mammographic images using the *Otsu's method*. |
| Region 5 | Finds the *dense* region in mammographic images using the *Iterative Otsu's method*. |
| Region 6 | Finds the *dense* region in mammographic images using the *arithmetic mean of pixels.* |
| Region 7 **(~)** | Finds the *fatty* region in mammographic images using our customized threshold (see Fig.S1(c) & Eq.S2) |
| Region 8 **(~)** | Finds the *fatty* region in mammographic images using the *entropy of the histogram*. |
| Region 9 **(~)** | Finds the *fatty* region in mammographic images using the *p-tile method* |
| Region 10 **(~)** | Finds the *fatty* region in mammographic images using the *Otsu's method*. |
| Region 11 **(~)** | Finds the *fatty* region in mammographic images usingthe *moment preserving thresholding method* |
| Region 12 | Finds the *dense* region in mammographic images using our customized threshold (see Fig.S1(c) & Eq.S2) |

**(~)** Denotes the logical “NOT” of a binary matrix. (*) CASAM-Area is calculated based on Region 6 in Eq.1 (in the main manuscript).
