# Supplementary material for: Area and Volumetric Density Estimation in Processed Full-Field Digital Mammograms for Risk Assessment of Breast Cancer
Source: PLoS One. 2014 Oct 20;9(10):e110690. doi: 10.1371/journal.pone.0110690 (PMC4203856; doi:10.1371/journal.pone.0110690)
Supplement: Algorithm S1 — A detailed description of the underlying algorithm for mammography image processing and segmentation. (DOCX) [file pone.0110690.s004.docx]

**Mammography Image Processing Algorithm**

*Pre-processing phase*

Prior to processing each mammogram we initially checked one of its tag fields (*APs.PhotometricInterpretation*) to determine whether the image’s greyscale needs to be inverted:

**if** isfield(APs, 'PixelIntensityRelationshipSign')

**if** (APs.PixelIntensityRelationshipSign==1)

Invert color;

**end**

**end**

The first proper step of our algorithm is to identify the “breast-to-air” region. Image segmentation has to fulfil certain predefined criteria and is one of the most difficult tasks in image processing [1]. Similar procedures that have been used for removing labels, markers and patient’s particulars in digitised film mammographic images are used here for reducing the size of FFDM images, by removing unnecessary black background. This step is carried out by identifying the vertical critical column, *j’,* in the mammographic image$\{I\in\Re^{2}\}$ of size$m\times n$. We use a vertical projection technique, of the intensity values, which is computationally undemanding yet very efficient in both FFDM and digitised film mammographic images. A vertical projection can be seen as a plot in which each index, in *b_j_*, corresponds to the sum of the pixels along column *j*.

$b_{j}=\int_{-\infty}^{\infty} I(i,j)dj$ (S1)

This integral can be approximated by summation ($b_{j}=\sum_{i=1}^{m} I(i,j)$). Then, locating the global minimum in the vector *b* yields the vertical critical column:$j'=\left\{j'/b_{j'}=\min(b)\right\},j\in\left[1,2,...,n\right]$. Similarly, the projection method is adopted to locate the horizontal bottom cut in the image (i.e., where the breast ends) as shown in Figure S1 (compare (a) to (b)). The image is then down-sampled to 512 pixels height using bilinear interpolation while preserving the image aspect ratio for the width. Next, the signal-to-noise ratio is improved by means of applying a global spatial contrast adjustment to the mammograms. Limits to contrast stretch are found by considering the non-black region after performing image intensity normalization.

*Detection of breast area*

To choose a threshold algorithm for detecting the breast boundary (object blob) we first examined the following 13 different threshold algorithms, in a small subset of images: *concavity method; triangle algorithm; entropy method; inter-means method; iterative inter-means; maximum likelihood (EM); mean method; median method; minimum error; iterative minimum error; moments method; p-tile method and Otsu's method*. We choose to use the *triangle algorithm* due to its invariance to intra-breast intensity variations, making it our choice for detecting breast boundaries. Our algorithm then estimates the area of the objects in the resulting binary image and retains the largest object. Whilst the latter step, removal of non-breast objects, exhibits a mild effect in FFDM images, it is of crucial importance in digitised film mammographic images where there are labels, calibration wedges and various other paraphernalia on a mammogram. The position and orientation of these labels are not standard. Belkhodja et al.[2] use the Otsu method and a Gaussian filter in their algorithm to mask out labels in digitised film images.

*Segmentation and pectoral muscle detection*

The next step is to invoke an image segmentation routine. Image segmentation can be defined as the partitioning of a given image into non-overlapping, constituent regions that are homogeneous with respect to some characteristic such as intensity or texture [1] Ch.10. A survey of the medical image segmentation literature is provided by [3]. In this step we use Delaunay-based image segmentation, [4] , to estimate the number of pixels with homogenous intensity. This segmentation is fully automated, fast and does not require the user to provide an initial estimate of the number of clusters. The technique resembles the dynamic thresholding method used for segmentation, but it differs in terms of divide and merge-decision making*.* A Delaunay triangulation (*DT*), described in detail in [5], is constructed in our case from a set of points which corresponds to the probability density function (PDF) of the image intensities. The outer boundary of a *DT* is simply the convex hull (CV) of the set of the feature points where vertices provide a direct access to intensity values to be used in image segmentation. Time complexity ($O(n\log n),n_{histogram\_bins}\leq2^{8}$) is considerably lower than other approaches such as region growing and the deformable contours/snakes algorithms.

Let *I* be the entire mammographic image. Image segmentation can be seen as a process that partitions *I* into *n* sub-regions (*I_1_, I_2_,…,I_n_*) based on a clear measure such as homogeneity in such a way that regions in the segmented image (*S*) comply to:

$$\bigcup_{i=1}^{n} I_{i}=I$$

2. $I_{i}\bigcap I_{j}=$Ø$,\forall i\neq j.$
3. *I_i_* is a connected set, *i=1, 2…n*.

After this segmentation step, a pseudo-colour pattern (Red (R), Green (G), Blue (B)) is created using the composite of the original mammogram and its segmented version (*S*). We choose (arbitrarily) to represent the original image with the *R* component, in the RGB primary colour space. While the segmented image is treated as the *G* component in the native colour space, the *B* component is zeroed out (black here) to eliminate third component interference. Different perturbations can obviously be obtained. However, our choice is to have dense area (pectoral muscle and fibroglandular tissue) marked with a green gamut and the radiolucent tissue marked as red-orange; see the pseudo-colour pattern in Figure S1 (b). We examined two colour spaces namely, HSV and YIQ. It is interesting to note that *I* and *Q* components in the YIQ colour space have a very appealing property, namely that they provide an excellent separation of the red-orange colour from the green gamut. The HSV, on the other hand, is non-linear, therefore it is more computationally demanding than the YIQ transformation, especially in analyses of large volumes of medical data. The breast area segmentation step can be written in a simplified form based on the following signum function.

$sign(Q(x,y))=\left\{\begin{matrix} - \\ 0 \\ + \end{matrix}\right.\begin{matrix} : \\ : \\ : \end{matrix}\begin{matrix} Q(x,y)\in fatty\_region\_foreground \\ Q(x,y)\in image\_background \\ Q(x,y)\in Dense\_tissue\_foreground \end{matrix}$ (S2)

See Figure S1 (c, e) for an example of the application of the signum function to real mammographic data.

In order to remove all or most of the pectoral muscle, a mask needs to be created. Unlike existing methods where the pectoral muscle mask is constructed under the assumption that its boundary can be approximated with a single straight line on the top left corner for LMLOs, we propose the use of the convex hull allowing for fragmented straight lines. To create the mask, the smallest convex polygon (convex hull) is constructed from the binary image,$Q(x,y)<0$ (see, Figure S1 (c-d)).

The accurate detection of breast and pectoral muscle boundaries is a challenging task since in some cases the latter’s intensity blends together with the breast area where it becomes hard even for an observer to identify the border of the pectoral muscle. We therefore wanted to compare our approach in this step to alternative existing methods. A common approach is to use active contours (ACs), also known as Snakes or deformable contours [6] p. 299. Among those studies which used ACs in conjunction with mammography are [7,8], and the algorithm was recently evaluated in [9]. The ACs approach is based on an ingenious process that forms a set of points which aim to enclose a target shape, the shape to be extracted. The ACs approach works perfectly well in retrieving the true boundary of a fuzzy shape, thanks to its internal efficient energy minimization algorithm, making it an ideal option for semi-automatic processes. However, it fails to converge when the contour is tangent to the velocity vector and it can deform around a non-targeted object. For this reason the initialization phase can impact highly on the final result. Therefore, to enable a fair comparison with our developed algorithm, we assisted ACs to overcome these obstacles. We compared the result from our algorithm and the ACs approach in terms of the estimates of area PD to which they lead and against a third estimate (ground truth) of PD (obtained by the semi-automated approach Cumulus in a subset of 39 FFDM images. The Pearson correlation coefficients (correlation with Cumulus PD) were estimated as *r*=0.90 and *r*=0.79 for our PD proposed approach and the active contour algorithm, respectively. We measured time complexity on the larger dataset of 1011 processed FFDM images. The average time complexity to process a single image was recorded as mean=0.2712 sec and mean= 8.1614 sec for our PD proposed approach and the active contour algorithm, respectively. Both procedures are fast, although our approach can lead to remarkable time gain when considering a large volume of images as will be necessary for our study, the KARMA cohort. For instance, to process 190,000 images (the number of images which have currently been collected within KARMA) would take in excess of two weeks using the active contour algorithm as compared to merely 15 hours using our proposed approach.

Once the image of the breast has been segmented, we enhance the local contrast to reveal features present in the breast by applying contrast limited adaptive histogram equalisation (CLAHE) with the number of tiles being equal to (8×8). We have shown in previous studies pertaining to diabetes that CLAHE is a vital input for many imaging based medical oriented sciences; for further insights into its micro-biology applications see [10,11]. CLAHE is described mathematically as follows. Let *I* be the image with the segmented breast, the CLAHE transform $\bar{h}$is given by:

$\bar{h}(I)=[I_{\max}-I_{\min}]\rho(f)+I_{\min}$ (S3)

where$\rho(f)$represents the CDF (cumulative distribution function) denoted by $\rho(f)=\rho(X\leq x)=\int_{0}^{x} f(t)dt$.

**References:**

1. Gonzalez RC, Woods RE (2008) Digital Image Processing. Pearson.

2. Belkhodja L, Benamrane N (2009) Approche d’extraction de la région globale d’intérêt et suppression des artefacts radiopaques dans une image mammographique. Symposium international: images, multimédias, applications, graphiques et environnements, IMAGE’2009. Biskra, Algeria. pp. 239-248.

3. Pham DL, Xu C, Prince JL (2000) Current methods in medical image segmentation. Annu Rev Biomed Eng 2: 315-337.

4. Cheddad A, Mohamad D, Manaf AA (2008) Exploiting Voronoi diagram properties in face segmentation and feature extraction. Pattern Recognition 41: 3842-3859.

5. Costa L, Cesar R (2001) Shape Analysis and Classification: CRC Press.

6. Nixon M, Aguado A (2012) Feature Extraction & Image Processing for Computer Vision. Academic Press.

7. Wirth MA, Stapinski A (2003) Segmentation of the breast region in mammograms using active contours. Proceedings of the SPIE visual communications and image processing 515:1995-2006.

8. Ferrari RJ, Rangayyan RM, Desautels JE, Borges RA, Frère AF (2004) Identification of the breast boundary in mammograms using active contour models. Med Biol Eng Comput 42: 201-208.

9. Berks M (2010) Statistical models for synthesising the appearance of mammographic masses. PhD Thesis, The University of Manchester. Available: http://ethos.bl.uk/OrderDetails.do?uin=uk.bl.ethos.517844. Accessed 29 September 2014.

10. Cheddad A, Svensson C, Sharpe J, Georgsson F, Ahlgren U (2012) Image processing assisted algorithms for optical projection tomography. IEEE Trans Med Imaging 31: 1-15.

11. Hörnblad A, Cheddad A, Ahlgren U (2011) An improved protocol for optical projection tomography imaging reveals lobular heterogeneities in pancreatic islet and β-cell mass distribution. Islets 3: 204-208.
